# Marking of Tense and Agreement in Language Samples by Children With and Without Specific Language Impairment in African American English and Southern White English: Evaluation of Scoring Approaches and Cut Scores Across Structures

**DOI:** 10.1044/2020_JSLHR-20-00243

**Published:** 2021-01-20

**Authors:** Janna B. Oetting, Andrew M. Rivière, Jessica R. Berry, Kyomi D. Gregory, Tina M. Villa, Janet McDonald

**Affiliations:** a Department of Communication Sciences and Disorders, Louisiana State University, Baton Rouge; b Sentara, Woodbridge, VA; c Speech Pathology and Audiology Department, South Carolina State University, Orangeburg; d Department of Communication Sciences and Disorders, Pace University, New York, NY; e Teaching English to Speakers of Other Languages, Northeastern Illinois University, Chicago; f Department of Psychology, Louisiana State University, Baton Rouge

## Abstract

**Purpose:**

As follow-up to a previous study of probes, we evaluated the marking of tense and agreement (T/A) in language samples by children with specific language impairment (SLI) and typically developing controls in African American English (AAE) and Southern White English (SWE) while also examining the clinical utility of different scoring approaches and cut scores across structures.

**Method:**

The samples came from 70 AAE- and 36 SWE-speaking kindergartners, evenly divided between the SLI and typically developing groups. The structures were past tense, verbal –*s,* auxiliary BE present, and auxiliary BE past. The scoring approaches were unmodified, modified, and strategic; these approaches varied in the scoring of forms classified as nonmainstream and other. The cut scores were dialect-universal and dialect-specific.

**Results:**

Although low numbers of some forms limited the analyses, the results generally supported those previously found for the probes. The children produced a large and diverse inventory of mainstream and nonmainstream T/A forms within the samples; strategic scoring led to the greatest differences between the clinical groups while reducing effects of the children's dialects; and dialect-specific cut scores resulted in better clinical classification accuracies, with measures of past tense leading to the highest levels of classification accuracy.

**Conclusions:**

For children with SLI, the findings contribute to studies that call for a paradigm shift in how children's T/A deficits are assessed and treated across dialects. A comparison of findings from the samples and probes indicates that probes may be the better task for identifying T/A deficits in children with SLI in AAE and SWE.

**Supplemental Material:**

https://doi.org/10.23641/asha.13564709

Speech-language pathologists need to assess and treat children with specific language impairment (SLI) in dialect-appropriate ways. Unfortunately, a clinician's ability to do this well is dependent on a child's dialect. In mainstream dialects of English, such as General American English (GAE), all aspects of a child's linguistic system, including the marking of tense and agreement (T/A), can be assessed and treated. This is not the case for children who speak nonmainstream dialects of English, such as African American English (AAE) or Southern White English (SWE). Within these dialects, children's marking of T/A is best known as reflecting a language difference (from GAE) rather than a language disorder. Thus, to assess and treat T/A structures when working with an AAE-, SWE-, or other nonmainstream English–speaking child is to run the risk of misinterpreting the child's dialect as disordered.

At the same time, children with SLI who speak AAE, SWE, or other nonmainstream dialects of English deserve to have their entire language systems considered within clinical practice just as the entire systems of children who speak GAE are considered. In fact, we would argue that if the entire language systems of all children are not assessed and treated, as clinicians, we are engaged in a form of disparate practice. In recent years, we have also become more aware of the GAE-centric language that frames the *dialect versus disorder* phrase within our profession. As shown above, notice how the *dialect versus disorder* phrase is interpretable only when GAE serves as the gold standard. Without GAE as the reference, there are no dialectal differences to discuss when working with a speaker of nonmainstream English. Although studies of dialect differences are informative, when providing clinical services to young children learning a nonmainstream dialect of English, the focus should not be on how their dialect differs from GAE but on how well they are learning the forms, structures, and functions of their linguistic input relative to community-based and dialect-appropriate benchmarks.

As an alternative, our lab works from a *disorder within dialects* framework to guide research and clinical practice (Oetting, 2018; Oetting, Gregory, & Rivière, 2016; Oetting, McDonald et al., 2016). This framework is cross-linguistic and seeks to understand SLI within the context of various dialects, such as AAE and SWE, just as others seek to understand this same clinical condition in French, Italian, and Japanese (Leonard, 2014). This framework allows all aspects of AAE and SWE to be explored as a linguistic system (Green, 2011; Johnson & Koonce, 2018) and requires studies of SLI to use same dialect–speaking typically developing (TD) controls as the reference standard.

Our studies of child AAE and SWE are conducted in Louisiana, a state considered part of the Deep South. Relative to the rest of the county, the Deep South reports the highest levels of poor health and educational outcomes of its residents and children (Goldhagen et al., 2005; U.S. Department of Agriculture, 2017; Wood, 2003). We also focus many of our studies on children who live in rural areas, because AAE and SWE as spoken by adults in the rural South have been well documented and can be used as a guide (Bernstein et al. 1997; Wolfram, 1991; Wolfram & Ward, 2006).

As rural dialects in contact, child AAE and SWE contain many (but not all) of the same English forms (Oetting, 2019). For example, both dialects have *a, an,* and *the* as overt forms for articles (e.g., *I see a dog*); however, AAE child speakers may produce the article *a* when the following noun starts with a vowel, whereas SWE child speakers are more likely to produce the article *an* (e.g., *a orange* vs. *an orange*). Both dialects also have dialect-specific overt forms, such as *ain't* as an auxiliary, and both allow multiple morphemes to mark negation within an utterance (e.g., *I don't got none*). Finally, both dialects have several zero forms, such as the zero auxiliary form that can be used in second-person BE contexts (e.g., *You Ø walking*).

AAE-speaking children typically produce larger inventories and higher densities (i.e., frequencies) of nonmainstream overt forms and zero forms than SWE-speaking children, although in both dialects, there are individual differences, with some children producing low, medium, and high densities of these nonmainstream forms (Oetting & McDonald, 2002; Seymour et al., 2003; Van Hofwegen & Wolfram, 2010; Washington & Craig, 1994). Child AAE and SWE also differ from each other in the linguistic contexts in which various mainstream and nonmainstream forms are preferred (Roy et al., 2013) and in their use of some forms for dialect-specific functions. These forms are referred to as camouflaged, because they are produced across dialects of English, including GAE, but they serve expanded grammatical, semantic, or pragmatic functions in AAE and/or SWE (Blake & Buchstaller, 2020; Green, 2002; Lanehart, 2015). For example, both dialects allow past participle forms (e.g., *seen*) to be used as markers of the simple past (i.e., *I seen it*). Child AAE has a larger inventory of camouflaged forms than child SWE, and all camouflaged forms are vulnerable to misinterpretation if a GAE lens is used to evaluate their grammaticality. Through a GAE lens, a camouflaged form like *seen* is an error, whereas through an AAE or SWE lens, this form reflects a dialect-appropriate overt form expressing the simple past. Another camouflaged form relevant to the current study is the preterite had + verb form as in, *He dropped it. Then he had picked it up*. As shown by this example, *had picked* expresses the simple past rather than the past perfect. This form does not require the second verb to be overtly marked for past tense (i.e., *had picked* and *had pick* are grammatical), and its use in adult and child AAE is well documented (e.g., Craig & Grogger, 2012; Rickford & Rafal, 1996; Ross et al., 2004).

Previous SLI studies using a *disorder within dialects* framework have revealed three important findings regarding the marking of T/A in AAE and SWE: (a) Children with and without SLI express T/A using a large and diverse inventory of mainstream overt forms (e.g., *cried, saw, fell)*, nonmainstream overt forms (e.g., *had cried, had cry, kickeded, seen, falled,* verbal –*s* with first- and second-person subjects), and nonmainstream zero forms (e.g., *cryØ, seeØ, fallØ*); (b) the relative frequencies at which these various forms are produced vary by a child's dialect (AAE vs. SWE) and T/A structure (e.g., past tense vs. verbal –*s*)^1^; and (c) AAE- and SWE-speaking children with SLI often produce lower percentages of mainstream and nonmainstream overt forms and higher percentages of nonmainstream zero forms than same dialect–speaking TD controls (for a review of language sample studies, see Oetting, 2019; see also Hendricks & Adlof, 2020; Smith & Bellon-Harn, 2015).

Recently, Oetting et al. (2019) developed four dialect-informed probes to examine the T/A systems of AAE- and SWE-speaking kindergartners. The probes elicited eight T/A structures (i.e., regular and irregular past tense, habitual and nonhabitual verbal –*s,* auxiliary BE present *is* and *are,* auxiliary BE past *was* and *were*), and each probe included 16 items (eight per structure; total = 64). Although overt and zero forms are grammatical in AAE and SWE, the probes were designed to encourage overt marking, which we reasoned would maximize differences between the SLI and TD groups' percentages of overt forms.

The probes also allowed for an evaluation of three scoring approaches (i.e., unmodified, modified, and strategic) and two types of cut scores (i.e., dialect-universal and dialect-specific). As shown in Table 1, the unmodified scoring approach mirrored those used in most GAE-centric tests by considering only mainstream overt forms as correct and all other responses as incorrect.^2^ The modified approach mirrored those recommended for most GAE-centric tests when the child being tested speaks a nonmainstream dialect of English. This approach considered the mainstream and nonmainstream overt forms and zero forms as dialect appropriate and correct and all other responses as incorrect. Finally, the strategic scoring approach considered the mainstream and nonmainstream overt forms and nonmainstream zero forms as dialect appropriate and correct but divided the sum of both types of overt forms by the sum of the overt forms and zero forms, just as one might do when calculating children's percentages of noun subjects out of their total noun and pronoun subjects. Unlike the other two approaches, this approach excluded all responses that could not be coded as one of these three form types, as the focus was on the children's relative use of overt forms and not on their ability to complete an item (i.e., *I don't know* responses or responses without a verb were excluded). Evaluating different approaches to scoring is critical as they impact clinical decisions about a child's language abilities. Modified scoring approaches have also been a mainstay in the pedagogy of speech-language pathologists when nonmainstream dialect speakers are discussed, yet these approaches have rarely been evaluated for their validity (Hendricks & Adlof, 2017).

**Table 1.** Description of the three scoring approaches with examples for the past tense form of *eat* (Oetting et al., 2019).

Approach	Description and Examples	
Unmodified	Correct/(Correct + Incorrect)	
	Correct	Mainstream overt forms (e.g., *ate*)
	Incorrect	All other responses (e.g., *eatØ, ated, had ate, had eat, eats, was eating, were eating, the boy, no response, I don't know*)
Modified	Correct/(Correct + Incorrect)	
	Correct	Mainstream and nonmainstream overt forms and zero forms (e.g., *ate, eatØ, ated, had ate, had eat*)
	Incorrect	All other responses (e.g., *eats,* was eating, *were eating, the boy, no response, I don't know*)
Strategic	Correct Overt/(Correct Overt + Correct Zero)	
	Correct overt	Mainstream and nonmainstream overt forms (e.g., *ate, ated, had ate, had eat*)
	Correct zero	Nonmainstream zero forms (e.g., *eatØ*)
	Excluded	All other responses (e.g., *boy, eats, was eating, were eating, no response, I don't know*)

A cut score within assessment refers to the value on a test or measure that is used to classify a child as presenting or not presenting a clinical weakness (Dollaghan, 2007). Cut scores are often articulated in standard deviation units, such as −1.0, −1.5, or −2.0 *SD*s from the normative mean, but they can also be criterion-referenced, as implemented on the Test of Early Grammatical Impairment (TEGI; Rice & Wexler, 2001). Within the probe study, the dialect-universal method relied on a single, criterion-based score to classify children as presenting or not presenting a T/A weakness ignoring the dialect they spoke, whereas the dialect-specific method allowed for different criterion-based scores to be used based on a child's dialect. Although dialect-universal cut scores are the norm within clinical practice, both the MacArthur–Bates Communicative Development Inventories (Fenson et al., 2007) and the Goldman-Fristoe Test of Articulation (Goldman & Fristoe, 2000) offer separate norms (and separate cut scores) for boys and girls. Thus, the use of multiple cut scores in clinical practice is not without precedence.

Results from the probe study showed the strategic scoring approach to best differentiate the SLI and TD groups within both dialects while minimizing differences between the children's AAE and SWE dialects. In addition, the highest levels of SLI versus TD classification accuracy were found when the cut scores were dialect-specific, and the best absolute classification accuracy level was found for dialect-specific cut scores on items that elicited irregular past tense. For these items, the optimal cut score was 50% for AAE and 71% for SWE, and these cuts led to a classification accuracy of 76% (Se = .74, Sp = .77) in AAE and 89% (Se = .89, Sp = .89) in SWE. Finally, the probes revealed dialect-specific patterns of overt marking for the TD groups that were consistent with the adult AAE and SWE literature. That is, the AAE TD group produced higher percentages of mainstream and nonmainstream overt forms for past tense than for verbal –*s,* and for *was* and we*re* than for *is* and *are,* whereas the SWE TD group produced relatively high percentages of mainstream and nonmainstream overt forms for past tense, verbal –*s, was, were,* and *is,* with a lower percentage produced for *are*. The SLI groups also presented dialect-specific patterns of marking that were consistent with the TD controls, but the differences were less dramatic due to their overall lower percentages of overt forms.

Although results from the probe study support a clinical focus on T/A across dialects, they also highlight the need to tailor measures of these structures to a child's dialect. Based on the probe results, this means examining a wide range of T/A structures, recognizing the full inventory of dialect-appropriate mainstream and nonmainstream overt forms and zero forms, along with strategic scoring of these forms, and using dialect-specific cut scores to guide clinical decisions about the relative strengths or weaknesses of a child's grammar. The current study was designed to examine the validity of these recommendations by testing the generalization of the probe findings using language samples and measures of the same T/A structures. Compared to the formal testing format of probes, samples are more likely to capture children's natural use of language when they are conversing with others at school (Stockman, 1996; Stockman et al., 2016). If the probe results do not generalize to children's natural use of language, it may be premature to recommend strategic scoring, dialect-specific cut scores, or even a focus on T/A when working with AAE- and SWE-speaking children with SLI. However, if findings from the probes generalize, there will be additional evidence to support the inclusion of T/A measures within clinical practice and new information about how best to do this for children with SLI who speak a dialect other than GAE.

Findings reported by Rudolph et al. (2019) illustrate the need for rigorous study of the methods used to measure T/A when working with diverse groups of English dialect speakers. Their participants were 676 six-year-olds (67% White, 30% African American, 3% other/indeterminate); the data were language samples that had been elicited by caregivers; and the T/A measure was a composite score that combined regular past tense, verbal –*s*, and auxiliary and copular *am, is,* and *are*. Although the authors made two minor scoring modifications for nonmainstream English forms, they did not classify the children's dialects, consider the children's full inventory of dialect-appropriate forms, or utilize dialect-specific cut scores, even though the best predictor of the children's percentages of mainstream overt marking for the T/A structures were those of their caregivers (which likely indicated that the children were producing the T/A forms at percentages consistent with their caregivers' dialects). The authors also included samples that contained low numbers of T/A forms, with at least one sample including just one form. Finally, the authors used a single cut score of 85% for the children's T/A scores, which came from studies of GAE (Gladfelter & Leonard, 2013; Rice & Wexler, 2001).^3^ Not surprisingly, classifications made with the GAE cut score for the children's T/A percentages did not align well with their own empirically derived cuts made for the children's mean length of utterance (MLU) levels or scores from two language tests. Although the authors interpreted their findings as not supporting measures of T/A when working with diverse groups of English dialect speakers, it may have been their methods for measuring and interpreting T/A that were inappropriate. In the current study, we investigate this possibility by examining different scoring approaches, cut scores, and T/A structures.

## Summary and Research Questions

Dialect-informed probes targeting T/A hold promise for clinical practice when working with AAE- and SWE-speaking children, but more work is needed to examine how well results from probes generalize to children's use of language when they are naturally interacting with others at school. Using language samples from the same AAE- and SWE-speaking children who participated in the probe study, we asked the following questions:

1. Do children produce a large and diverse inventory of mainstream and nonmainstream overt forms and zero forms to mark T/A structures within their language samples, and are the frequency distributions of these forms similar to what was previously found for probes?
2. As was found for the probes, does a strategic scoring approach relative to two other scoring approaches lead to the largest within-dialect differences between the SLI and TD groups, while also reducing differences between the children's AAE and SWE dialects?
3. Compared to a single, dialect-universal cut score, do dialect-specific cut scores lead to higher levels of SLI versus TD classification accuracy within AAE and SWE?
4. Of the T/A structures, do measures of past tense lead to the highest levels of SLI versus TD classification accuracy?

## Method

### Participants

Participants were 106 kindergartners studied by Oetting et al. (2019). Data collection occurred after institutional review board approval, caregiver consent, and child assent, and all data were collected at the children's schools.

Table 2 lists summary information about the participants. All children lived in the rural South and attended public schools. Seventy were African American and classified as speakers of AAE, and 36 were non–African American and classified as speakers of SWE, with equal numbers of children within each dialect classified as SLI and TD. Dialect classifications were based on the children's responses on Part I of the Diagnostic Evaluation of Language Variation–Screening Test (Seymour et al., 2003) and blind listener judgments of 1-min conversational excerpts from the children, with the children's full language samples explored in a few cases. The Diagnostic Evaluation of Language Variation–Screening Test Part I quantified the density of children's nonmainstream form use within their responses; whereas the listener judgment task classified the children's type of dialect as AAE, SWE, or other (for a comparison across tasks, see Oetting et al., 2019).

**Table 2.** Participant profiles by dialect and clinical status.

Variable	AAE		SWE	
	SLI (*n* = 35)	TD (*n* = 35)	SLI (*n* = 18)	TD (*n* = 18)
Age^a^	66.94 (3.74)	65.60 (3.55)	65.72 (3.89)	66.61 (4.18)
Maternal education^b^	11.67 (2.27)	13.27 (2.62)	12.33 (2.87)	13.17 (3.05)
PTONI^c^	93.69 (9.62)	98.09 (8.87)	96.50 (8.35)	98.28 (8.14)
GFTA-2^d^	104.49 (5.72)	107.00 (4.38)	104.78 (4.18)	110.50 (3.09)
DELV–Norm Referenced Syntax^e^	4.83 (1.01)	10.00 (1.55)	4.78 (1.67)	10.39 (1.72)
PPVT-4^f^	82.34 (9.42)	101.06 (9.32)	85.78 (7.01)	105.56 (5.6)
C&I utterances^g^	243.31 (57.80)	230.94 (55.15)	255.89 (72.19)	216.72 (60.56)
MLU^h^	5.53 (.99)	6.46 (1.0)	5.13 (.86)	6.92 (1.05)
% Utterances with nonmainstream forms^i^	36.9 (10.54)	32.5 (09.35)	28.5 (15.12)	15.08 (04.8)

*Note.* Data are reported as mean (standard deviation). Reprinted with modification from Oetting et al. (2019). AAE = African American English; SWE = Southern White English; SLI = children with specific language impairment; TD = typically developing children; PTONI = Primary Test of Nonverbal Intelligence; GFTA-2 = Goldman-Fristoe Test of Articulation–Second Edition; DELV–Norm Referenced = Diagnostic Evaluation of Language Variation-Norm Referenced; PPVT-4 = Peabody Picture Vocabulary Test–Fourth Edition; C&I = complete and intelligible utterances; MLU = mean length of utterance.

a Age in months.

b Years of schooling (i.e., 12 high school graduates, with data missing for four children).

c Standardized scores for the PTONI (normative *M* = 100, *SD* = 15).

d Standardized scores for the GFTA-2 (normative *M* = 100, *SD* = 15).

e Standardized scores for the Syntax subtest of the DELV–Norm Referenced (normative *M* = 10, *SD* = 3).

f Standardized scores for the PPVT-4 (normative *M* = 100, *SD* = 15).

g Number of C&I analyzed in samples.

h MLU in morphemes.

i Percentage of utterances within sample with nonmainstream English forms.

The children's clinical status (SLI or TD) was determined through a battery of standardized tests, and each child with SLI was matched to a TD control based on dialect spoken, age, and nonverbal IQ score, and then, as much as it was possible, maternal educational level. As noted in previous studies, 45% of the SLI group and 15% of the TD group presented a positive family history of speech, language, or reading impairment. Also, the 53 children classified as SLI reflected 6% of the 834 kindergartners within the schools and 8% of the 669 kindergartners with signed consent forms.

Children in both groups passed a hearing screening, performed at or above −1.2 *SD*s of the normative mean on the Primary Test of Nonverbal Intelligence (Ehrler & McGhee, 2008) and above −1 *SD* of the normative mean on the Goldman-Fristoe Test of Articulation (Goldman & Fristoe, 2000). Children in the SLI group performed at or below −1 *SD* of the normative mean on the syntax portion of the Diagnostic Evaluation of Language Variation–Norm Referenced (Seymour et al., 2005), whereas those in the TD group performed above this cutoff. For descriptive purposes, all children also completed the Peabody Picture Vocabulary Test–Fourth Edition (Dunn & Dunn, 2007) and the phonological screener from the TEGI (Rice & Wexler, 2001). The screener includes 20 items, with five targeting /s, z, t/ and /d/ in the word-final position. All children passed the screener by accurately producing the target or a phonologically consistent approximation on four out of five items for each sound.

### Materials

#### Language Samples

Samples were elicited by having each child and an examiner play together for 20–30 min. Materials included toys (i.e., a gas station set, picnic/park set, and baby doll set) and three action pictures (i.e., a child at a doctor's office getting a shot and a family fishing, grocery shopping, or washing a car; Arwood, 1985). The sessions started with the toys, which were introduced in the order of the children's preferences. During the sessions, the examiners followed the children's leads and used prompts (e.g., *I bet you've…*, *I wonder what happened when…*) to encourage the children to talk about past personal experiences, and they produced back channeling (e.g., *uhhuh, wow, no way, really*) and affirmative comments (e.g., *that's amazing, that sounds like it was fun, you were so brave*) to encourage the children to continue talking. Toward the end of each session, the examiners showed the children a picture while modeling a story and then asked the children to tell their own stories with pictures used as prompts.

Each sample was audio-recorded and later transcribed and coded using WavPedal software (WavPedal.com) and Systematic Analysis of Language Transcripts software (Miller & Iglesias, 2018). Transcription and coding of the samples included at least three passes by at least two research assistants. Transcription and coding followed procedures outlined in Oetting et al. (2014), which allows utterances to contain two but not three conjoined independent clauses and all associated modifiers and dependent clauses. Only complete and intelligible utterances within the samples were analyzed; these utterances totaled 25,106 and averaged 236.85 (*SD* = 60.51) per child. For descriptive purposes, the children's number of complete and intelligible utterances, MLU in morphemes, and percentage of utterances with nonmainstream English forms (% nonmainstream) are listed in Table 2. Although we attempted to collect equal numbers of utterances from the children, the examiners spent more time eliciting the SLI samples than the TD samples, and the SLI samples ended up slightly longer (SLI, *M* = 247, *SD* = 63.00 vs. TD, *M* = 226, *SD* = 56.87), *F*(1, 102) = 4.38, *p* = .039, η_p_^2^ = .04.^4^ For MLU, there also was a main effect for clinical group (SLI < TD), *F*(1, 102) = 45.97, *p* < .001, η_p_^3^ = .31, and an interaction between the children's clinical group and dialect, *F*(1, 102) = 4.59, *p* = .035, η_p_^2^ = .04, which related to a larger effect of the clinical difference in SWE, *F*(1, 34) = 31.40, *p* < .001, η_p_^2^ = .48, than in AAE, *F*(1, 68) = 15.53, *p* < .001, η_p_^2^ = .19.

For the percentage of utterances with nonmainstream forms, there were main effects for the children's dialect, *F*(1, 102) = 36.53, *p* < .001, η_p_^2^ = .26, and clinical group, *F*(1, 102) = 17.62, *p* < .001, η_p_^2^ = .15, and an interaction between these variables, *F*(1, 102) = 4.43, *p* = .038, η_p_^2^ = .04. As expected, the AAE speakers produced higher percentages of utterances with nonmainstream forms than did the SWE speakers, although the effect was larger for the TD group, *F*(1, 51) = 54.35, *p* < .001, η_p_^2^ = .52, than for the SLI group, *F*(1, 51) = 5.59, *p* = .022, η_p_^2^ = .10. In addition, the children's percentages of utterances with nonmainstream forms did not differ between the clinical groups in AAE, *F*(1, 68) = 3.51, *p* < .065, η_p_^2^ = .05, but it did in SWE, *F*(1, 34) = 12.91, *p* < .001, η_p_^2^ = .28.

### T/A Coding

Within the samples, codes were added to contexts that could support the T/A structures targeted in the probes (i.e., regular and irregular past tense, habitual and nonhabitual verbal –*s,* auxiliary BE present *is* and *are,* auxiliary BE past *was* and *were*). Regular and irregular past tense contexts were limited to those supporting main verbs (e.g., *brushed, saw*) but not participles (e.g., *was brushed, was seen*), auxiliaries (e.g., *have/had* or *do/did*), no change forms such as *cut, put,* and *hit*, nor the all-purpose verb *got.* Verbal –*s* contexts were limited to those supporting regular forms (e.g., *plays*). BE present and past contexts were limited to those supporting auxiliaries (i.e., *___ walking*) but not copulas (i.e., *___ happy*), infinitival clause *be* (e.g., *I want to be a nurse*), past participle verb *be* (e.g., *I've been to the store before*), nonmainstream *ain't, be_2_,* or *be_3_* (e.g., *We ain't working, We be working, We be Baton Rouge*; Spears, 2020), nor nonmainstream BIN (e.g., *They BIN left*; Green, 2002, 2011). Also, no nonmainstream forms of auxiliary BE past BEEN (e.g., *Yesterday, we* *BE* *EN swimming*), which have been documented in the language samples of AAE-speaking children with Gullah/Geechee heritage were found in the samples (Berry & Oetting, 2017).

The children's T/A contexts were coded as marked by either a mainstream overt form, nonmainstream overt form, or nonmainstream zero form. Mainstream overt forms were consistent with those typically listed in standard English resource books (e.g., *jumped, ate, drinks, is, are, was, were*). Nonmainstream overt forms included those documented in previous AAE and SWE studies. These included past tense expressed with dialect-specific forms (e.g., *drunk, seen*); double marked forms (e.g., *jumpeded*), overregularized forms (e.g., *ated*); and preterite had + verb forms (e.g., *had jumped, had jump, had fell, had fall*), verbal –*s* forms with first- and second-person singular or plural subjects (e.g., *I walks, You walks, They walks*), *is* with first- and second-person singular and plural subjects (e.g., *I's cooking, You's cooking, They's cooking*), *was* with second-person singular and plural subjects (e.g., *You was going, We was going*), *are* and *were* with singular third-person subjects (e.g., *He are playing, She were playing*), and overt forms of any of the T/A structures within noninverted *wh*-questions (e.g., *Where y'all went? How many soups she wants? What this is?*).^5^ Zero forms were identified when a T/A form was produced without perceived phonetic content in the surface structure of the utterance (e.g., *walkØ, bringØ, he Ø walking, they Ø running*).

The children also occasionally (approximately 1% of the time; *n* = 93 out of 8,732 coded forms) produced a T/A form in a context that was potentially inappropriate for the child's dialect. These included predicate contexts with more than one overtly marked T/A form (e.g., *where did this went, he's goes, they was started picking*) and overt marking of T/A within nonfinite predicate contexts (e.g., *they want to jumps*). In addition, the children produced some overt forms of verbal –*s* with the verb *got* in utterances without an expressed auxiliary *has* (e.g., *he gots it, she gots to do it*). These forms were coded as “other” (i.e., for a similar treatment of *got,* see Cleveland & Oetting, 2013). Of note, the previous probe study also included child responses coded as other; however, those responses were different than those coded as other in the samples (i.e., they included no responses and responses to a probe item that did not obligate the targeted T/A structure). For now, we note the task difference and revisit the nature of the other responses in the discussion.

### Scoring of T/A

Following the procedures used in the probe study, percentages of overt marking with unmodified scoring were calculated by dividing the sum of the children's mainstream overt forms by the sum of their coded forms. Percentages of overt marking with modified scoring were calculated by dividing the sum of the children's mainstream and nonmainstream overt forms and zero forms by the sum of their coded forms. Percentages of overt marking with strategic scoring were calculated by dividing the sum of the children's mainstream and nonmainstream overt forms by the sum of their mainstream and nonmainstream overt forms and zero forms. Like the probe study, strategic scoring did not include forms coded as other (see Supplemental Material S1 for a comparison of the three scoring approaches as applied to data from four children).

### Reliability

Reliability of language sample transcription and coding was checked using data from the 106 children included in the study and other samples from children who did not meet the current study's inclusionary and exclusionary criteria (for details of the larger data set, see Rivière et al., 2018). Reliability of the transcriptions was examined by having a second set of examiners independently transcribe and code the randomly selected, 1-min excerpts from the samples that were used in the listener judgment dialect task. The same three-pass transcription and coding system was followed for these excerpts. Between the samples and excerpts, the average rate of agreement for utterance boundary decisions, words transcribed, and morphemes coded was 93%, 95%, and 97%, respectively. We also checked the reliability of categorizing and summing the children's various forms for each T/A structure by asking a second team of examiners to independently categorize and sum the forms using a subset of the full samples (*n* = 50 samples or more, depending on the T/A structure). Across the T/A structures, agreement between examiners was 94% and ranged from 93% to 98%.

## Results

Following the probe study, analyses examined the children's coded forms with the T/A structures combined and then separated. For these analyses, we first examined the number and type of each coded form produced by the children. Then, using these forms, we calculated percentages of overt marking using the three scoring approaches and analyzed the unmodified and strategically scored data with analyses of variance (ANOVAs). As will be evident, modified scoring yielded ceiling effects for all groups, making statistical analyses not useful. ANOVAs were selected to directly compare the results to those from the probes; however, we also examined the data using logistic regression with a binomial distribution on the proportion of relevant forms calculated with the two scoring approaches (see Supplemental Material S2). Although these regressions identified more interactions than were found in the ANOVAs, the two statistical approaches led to similar conclusions about the clinical groups within AAE and SWE. The final step involved running discriminant function analyses with dialect-universal and dialect-specific cut scores to examine how well the T/A measures classified the children by their clinical status.

### T/A Structures Combined

**Figure 1.**
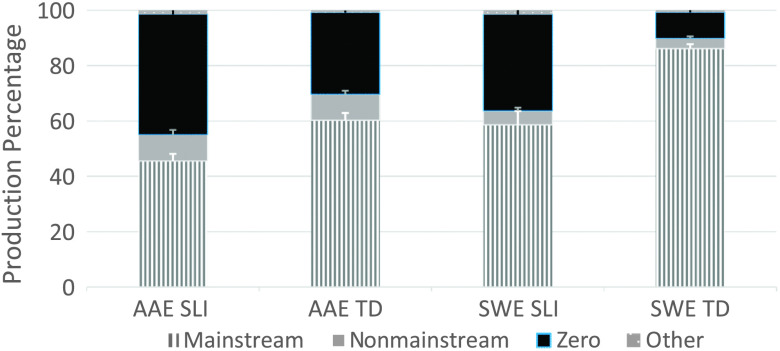
Tense and agreement form types by dialect and clinical status. AAE = African American English; SWE = Southern White English; SLI = children with specific language impairment; TD = typically developing children.

The total number of coded forms in the samples averaged 82.38 (*SD* = 29.15, range: 36–197, total = 8,732; see Table 3). Although the SLI samples were longer than the TD samples, a 2 (clinical group) × 2 (dialect) ANOVA indicated that the SLI group produced fewer coded T/A forms than the TD group, *F*(1, 102) = 4.86, *p* = .03, η_p_^2^ = .05. Nevertheless, all four child groups produced a large and diverse inventory of form types, with mainstream overt forms produced the most, followed by zero forms, nonmainstream overt forms, and then the other forms (see Figure 1). Of the four child groups, the SWE TD group produced the highest proportion of mainstream overt forms, the AAE TD group produced the highest proportion of nonmainstream overt forms, and the SLI groups produced the highest proportions of zero forms. Forms classified as other were rare for all four child groups.

**Table 3.** Number of coded forms by dialect and clinical status.

Variable	AAE		SWE	
	SLI	TD	SLI	TD
All structures	80.23 (26.55) 37–160	89.74 (34.64) 49–197	69.11 (19.75) 36–105	85.50 (26.55) 41–131

*Note.* Data are reported as mean, standard deviation (in parentheses), and range. AAE = African American English; SWE = Southern White English; SLI = children with specific language impairment; TD = typically developing children.

Using the children's coded forms, we calculated percentages of T/A marking using the three scoring approaches, and the results closely paralleled what was found for the probes (see Table 4). Percentages were lowest with unmodified scoring and highest with modified scoring, with the latter showing ceiling effects (*M* ≥ 98%) due to the low frequency of other forms. The strategic scoring approach led to percentages of marking that were in the middle of the other two approaches. When a 2 × 2 ANOVA was applied to the data, the unmodified approach showed clinical group and dialect differences as well as an interaction between these variables, which was related to a larger clinical group effect in SWE than in AAE (η_p_^2^ = .45 vs. .19) and a larger dialect effect for the TD group than for the SLI group (η_p_^2^ = .47 vs. .12). The strategic approach also showed a clinical group and dialect difference, but the interaction was only marginally significant, *F*(1, 102) = 3.55, *p* = .062, η_p_^2^ = .03. Moreover, the effect size of the clinical group difference with strategic scoring was the same as with unmodified scoring (η_p_^2^ = .30), but the effect size of the children's dialect difference was smaller (η_p_^2^ = .18 vs. .27), which is a desirable outcome.

**Table 4.** Mean (*SD*) percent marking by scoring approach, dialect, and clinical status.

Variable	AAE		SWE	
	SLI	TD	SLI	TD
Scoring approach				
Unmodified	46 (15)	60 (16)	58 (21)	86 (07)
Strategic	56 (14)	70 (13)	65 (24)	91 (06)
Modified	99 (02)	99 (01)	98 (03)	99 (01)
Significant effects				
Unmodified	Group, *F*(1, 102) = 44.40, *p* < .001, η_p_^2^ = .30 Dialect, *F*(1, 102) = 37.76, *p* < .001, η_p_^2^ = .27 Group × Dialect, *F*(1, 102) = 4.11, *p* = .045, η_p_^2^ = .04 SLI dialect, *F*(1, 51) = 6.68, *p* = .013, η_p_^2^ = .12 TD dialect, *F*(1, 51) = 45.78, *p* < .001, η_p_^2^ = .47 AAE group, *F*(1, 68) = 16.03, *p* < .001, η_p_^2^ = .19 SWE group, *F*(1, 34) = 27.87, *p* < .001, η_p_^2^ = .45			
Strategic	Group, *F*(1, 102) = 42.67, *p* < .001, η_p_^2^ = .30 Dialect, *F*(1, 102) = 22.36, *p* < .001, η_p_^2^ = .18			
Modified	NA			
Classification accuracy SLI vs. TD				
Unmodified	Cut score = 60%, Classification accuracy 67%, Se = .70, Sp = .64			
Strategic	Cut score = 67 or 68%, Classification accuracy 73%, Se = .72, Sp = .74			
Modified	Cut score = 98.9%, Classification accuracy 54%, Se = .43, Sp = .64			

*Note.* AAE = African American English; SWE = Southern White English; SLI = children with specific language impairment; TD = typically developing children; NA = not applicable.

Finally, discriminant analyses were completed to examine how well each scoring method classified the children by their clinical status. With the dialects combined, the strategic scoring approach led to the highest level (73%) of classification accuracy. The other two scoring approaches not only led to lower classification accuracies (67% and 54%), but the unmodified approach misclassified many children in the TD group as SLI (i.e., Sp = .64), and the modified approach misclassified many children in the SLI group (Se = .43) and the TD group (Sp = .64). When the children were separated by their dialects to allow for dialect-specific cut scores, classification accuracy with the strategic approach improved slightly to 74% (AAE cut score between 61.54% and 64.41%: 71% accuracy, Se = .71, Sp = .71; SWE cut score between 65.31% and 78.12%: 78% accuracy, Se = .56, Sp = 1.00). Interestingly, calculating these same cut scores by hand using the maximum (i.e., best discrimination) of Youden's *J* index led to even greater differences between the cut scores for the two dialects and even higher levels of classification accuracy (i.e., best AAE cut was 60%, overall accuracy = 73%, Se = .66, Sp = .80; best SWE cut = 80%, overall accuracy = 86%, Se = .72, Sp = 1.00).

### T/A Structures Separated

As shown in Table 5, the samples contained many coded forms for past tense irregular and relatively high numbers of coded forms for past tense regular and *is,* although even for these latter two structures, some samples contained no more than one to three coded forms. The numbers of coded forms for the other T/A structures were very low, with multiple samples including no forms or few forms. There were also differences in the number of forms by the children's clinical groups and dialects; clinical differences (SLI < TD) were found for irregular past, *F*(1, 102) = 7.058, *p* = .009, η_p_^2^ = .07, and *were*, *F*(1, 102) = 7.71, *p* = .007, η_p_^2^ = .07, and dialect differences (AAE > SWE) were found for *was, F*(1, 102) = 5.54, *p* = .021, η_p_^2^ = .05, and *were, F*(1, 102) = 4.87, *p* = .03, η_p_^2^ = .05.

**Table 5.** Number of coded forms for each tense and agreement structure by dialect and clinical status.

Variable	AAE		SWE	
	SLI	TD	SLI	TD
Past tense regular Σ = 1,447	13.34 (8.95) 3–50	15.83 (8.86) 5–39	10 (4.17) 4–19	13.67 (7.31) 5–30
Past tense irregular Σ = 2,809	24.97 (15.67) 10–85	29.34 (15.88) 9–92	19.50 (7.25) 9–35	30.94 (14.69) 7–55
Verbal –*s* habitual Σ = 1,048	9.43 (5.86) 0–24	9.86 (7.97) 1–38	10.44 (8.93) 1–38	10.28 (6.49) 1–24
Verbal –*s* nonhabitual Σ = 641	5.54 (4.95) 0–20	6.60 (7.36) 0–35	5.83 (4.68) 0–19	6.17 (4.88) 0–17
Auxiliary *is* Σ = 1,103	10.17 (6.47) 3–31	10.31 (7.37) 1–34	11.67 (10.64) 1–39	9.78 (9.12) 0–35
Auxiliary *are* Σ = 665	6.83 (5.04) 0–26	5.89 (4.09) 0–17	7.17 (6.86) 0–24	5.06 (4.09) 0–15
Auxiliary *was* Σ = 725	7.57 (5.65) 0–25	7.86 (5.58) 0–30	3.33 (3.33) 0–11	6.94 (5.80) 0–21
Auxiliary *were* Σ = 294	2.34 (2.31) 0–12	4.06 (3.50) 0–18	1.22 (1.66) 0–6	2.67 (2.87) 0–11

*Note.* Data are reported as mean, standard deviation (in parentheses), and range. AAE = African American English; SWE = Southern White English; SLI = children with specific language impairment; TD = typically developing children.

**Figure 2.**
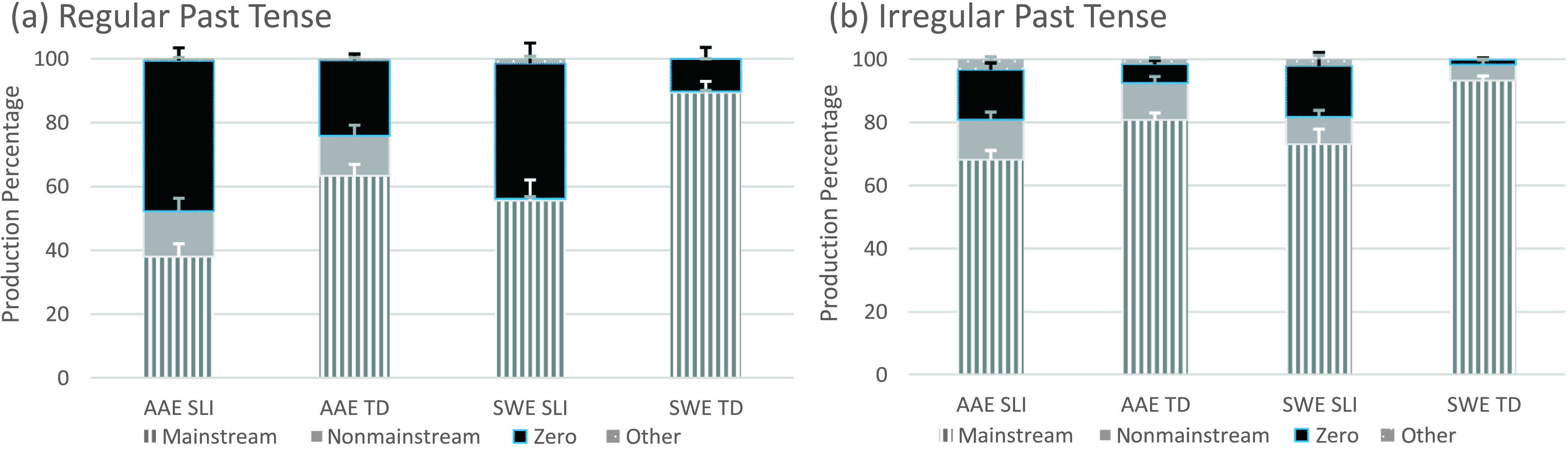
Past tense form types by structure, dialect, and clinical status. AAE = African American English; SWE = Southern White English; SLI = children with specific language impairment; TD = typically developing children.

As mentioned earlier, the eight T/A structures remained separated to examine the frequency distributions of the coded forms (see Figures 2 [Fig F3] [Fig F4]–5), as was done in the probe study; these results also included language samples from all children. However, given the low numbers of some of the coded forms in some of the language samples, statistical analyses of the individual T/A structures were limited to samples with at least eight coded forms, which was the number elicited on the probes. In addition, and again because of the low number of some of the coded forms, we collapsed the eight T/A structures into four (i.e., past tense, verbal –*s*, BE present, and BE past) for the statistical analyses. Had we not done this, over half of the samples would have been excluded for some of the analyses because of insufficient numbers of coded forms.

**Figure 3. F3:**
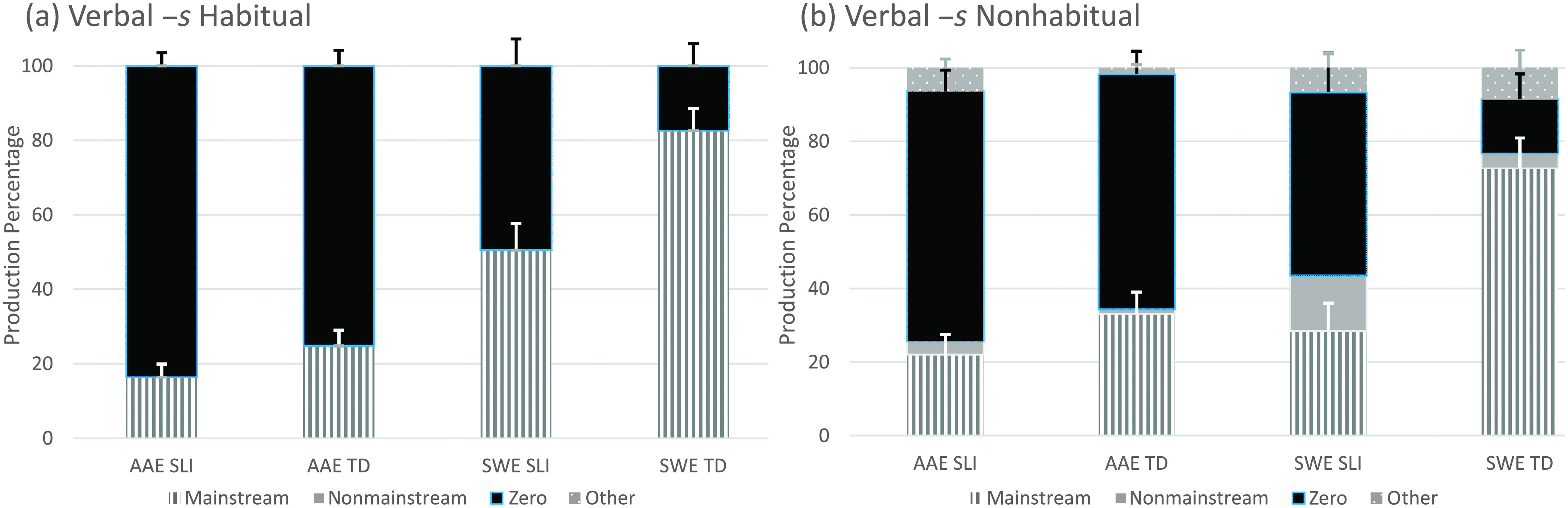
Verbal –*s* form types by structure, dialect, and clinical status. AAE = African American English; SWE = Southern White English; SLI = children with specific language impairment; TD = typically developing children.

**Figure 4. F4:**
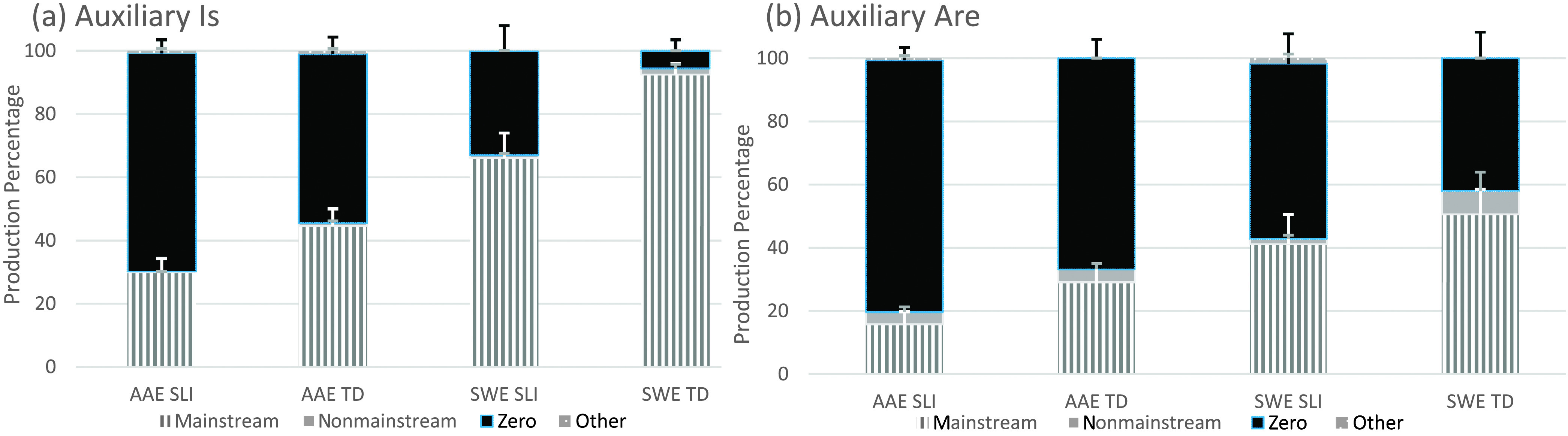
Auxiliary BE present form types by structure, dialect, and clinical status. AAE = African American English; SWE = Southern White English; SLI = children with specific language impairment; TD = typically developing children.

**Figure 5.**
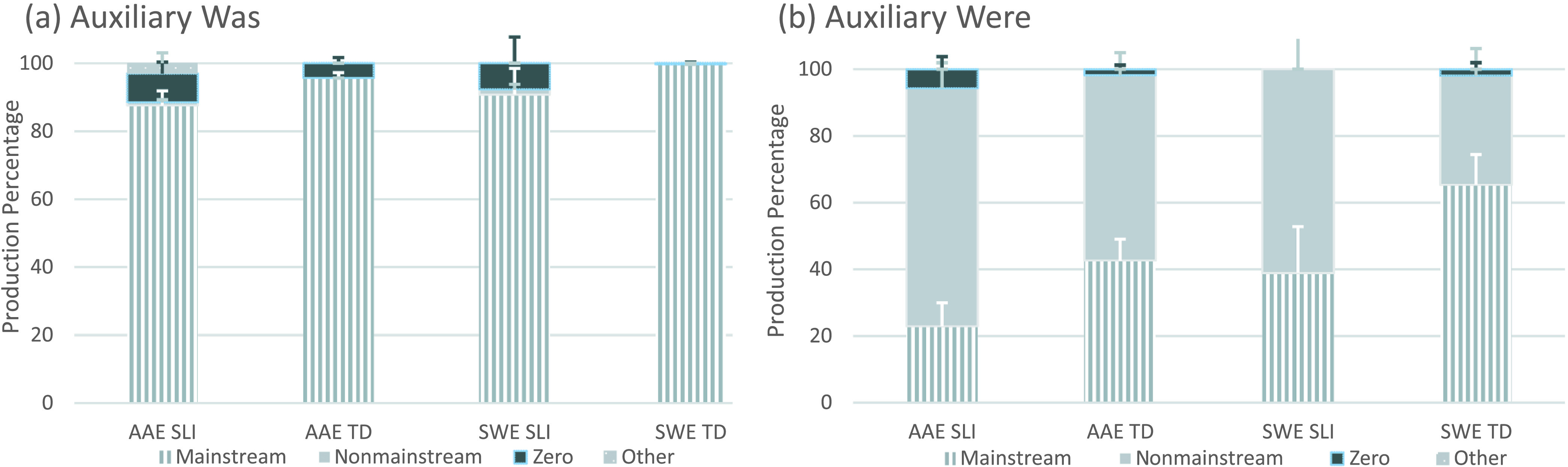
Auxiliary BE past form types by structure, dialect, and clinical status. AAE = African American English; SWE = Southern White English; SLI = children with specific language impairment; TD = typically developing children.

#### Past Tense

All four child groups produced all form types to express past tense (see Figures 2a and 2b). The highest proportions of mainstream overt forms were produced with irregular verbs, and the highest proportions of nonmainstream overt forms varied depending on the child's dialect. The AAE speakers produced nonmainstream overt forms with regular and irregular verbs, whereas the SWE speakers produced these forms primarily with irregular verbs. The nonmainstream overt regular forms included one double marked form (i.e., *She likeded it*) and 169 had + verb forms (e.g., *And his mama had whooped him, He had punch him*). The nonmainstream overt irregular forms included 98 overregularizations (e.g., *When he throwed the ball, She just catched the fish, I just leaved it alone*), 171 had + verb forms (e.g., *We had went to Ohio, He had shoot it across*), 23 dialect-specific irregular forms (e.g., *But guess what he brung me, Me and my daddy seen I think a mama alligator, We all drunk gatorade*), and 14 forms in a noninverted *wh*-question (e.g., *Where you went, What you did*). Zero forms were most frequently produced with regular verbs (e.g., *And then she pressØ the brake*), and other forms were rare (e.g., *I didn't wanted to eat it, She ain't trying to punched her, Did he ran over a fence or a dog*).

Percentages of past tense marking using the three scoring approaches were calculated using all 106 samples as each contained at least eight coded past tense forms when the regular and irregular verbs were combined. As shown in Table 6, unmodified and strategic scoring led to differences between the children's clinical groups and dialects, although the strategic approach led to a larger effect size of the clinical group difference (η_p_^2^ = .38 vs. .32) and a smaller effect size of the dialect difference (η_p_^2^ = .04 vs. .16). As before, statistical analyses were not applied to percentages from the modified scoring approach because of ceiling effects (all group *M*s ≥ 98%).

**Table 6.** Mean (*SD*) percent marking of past tense by dialect, clinical status, structure, and scoring approach.

Variable	AAE		SWE	
	SLI	TD	SLI	TD
Scoring approach				
Unmodified	58 (15)	75 (14)	67 (19)	92 (07)
Strategic	73 (13)	88 (06)	74 (18)	96 (05)
Modified	98 (04)	99 (01)	98 (04)	100 (00)
Significant effects				
Unmodified	Group, *F*(1, 102) = 49.40, *p* < .001, η_p_^2^ = .33 Dialect, *F*(1, 102) = 20.02, *p* < .001, η_p_^2^ = .16			
Strategic	Group, *F*(1, 102) = 62.91, *p* < .001, η_p_^2^ = .38 Dialect, *F*(1, 102) = 3.99, *p* = .048, η_p_^2^ = .04			
Modified	NA			

*Note.* AAE = African American English; SWE = Southern White English; SLI = children with specific language impairment; TD = typically developing children; NA = not applicable.

#### Verbal –s

All four child groups again produced all form types to express verbal –*s* (see Figures 3a and 3b). All groups except the SWE TD group produced the highest proportion of mainstream overt forms with nonhabitual forms and the highest proportion of zero forms with habitual forms. The SWE TD group showed the opposite pattern, although for both structures, this group produced high proportions of mainstream overt forms. Also, although all four child groups produced nonmainstream overt forms and other forms, proportions of both were higher for the SWE speakers than for the AAE speakers. Of the 28 nonmainstream overt forms, 19 were produced with plural subjects (e.g., *The red lights means stop, Me and my friend Randal plays with them*), two were produced with first-person subjects (e.g., *And now I sees right here*), and seven were produced within a noninverted *wh*-question (e.g., *How many soups she wants, Where this goes*). As was found for past tense, forms classified as other were rare, and 12 of these were with the verb *got* (e.g., *He gots a new bike*). Examples of forms classified as other that did not involve *got* included, *The boy is helping his dad washes his car, Does this goes right here,* and *Now he gonna goes in the store*.

Using the 89 (84%) samples containing at least eight coded verbal –*s* forms for the analyses, the unmodified scoring approach showed main effects for the children's clinical status and dialects and an interaction between these variables (see Table 7). Follow-up of the interaction indicated that a clinical difference (SLI < TD) was present in both dialects, but the effect size of the difference was larger in SWE than in AAE (η_p_^2^ = .39 vs. .11). Similarly, a dialect difference (AAE < SWE) was present in both clinical groups, but the effect size of the difference was larger in the TD group than the SLI group (η_p_^2^ = .52 vs. .32). The strategic approach also showed clinical group and dialect differences, but the interaction was only marginally significant, *F*(1, 85) = 3.57, *p* = .062, η_p_^2^ = .04. Moreover, the effect sizes of the differences with strategic scoring were comparable to those found with unmodified scoring (clinical group: η_p_^2^ = .20 vs. .24; dialect: η_p_^2^ = .45 vs. .43). Again, statistical analyses were not applied to percentages from the modified scoring approach because of ceiling effects (all group *M*s ≥ 97%).

**Table 7.** Mean (*SD*) percent marking of verbal –*s* by dialect, clinical status, and scoring approach (*n* = 89).

Variable	AAE		SWE	
	SLI (*n* = 29)	TD (*n* = 28)	SLI (*n* = 17)	TD (*n* = 15)
Scoring approach				
Unmodified	16 (16)	31 (26)	45 (27)	80 (17)
Strategic	18 (18)	32 (26)	52 (32)	85 (15)
Modified	97 (05)	99 (20)	97 (07)	97 (07)
Significant effects				
Unmodified	Group, *F*(1, 85) = 26.86, *p* < .001, η_p_^2^ = .24 Dialect, *F*(1, 85) = 64.24, *p* < .001, η_p_^2^ = .43 Group × Dialect, *F*(1, 85) = 4.42, *p* = .039, η_p_^2^ = .05 SLI dialect, *F*(1, 44) = 20.19, *p* < .001, η_p_^2^ = .32 TD dialect, *F*(1, 41) = 44.51, *p* < .001, η_p_^2^ = .52 AAE group, *F*(1, 55) = 7.00, *p* = .011, η_p_^2^ = .11 SWE group, *F*(1, 30) = 18.80, *p* < .001, η_p_^2^ = .39			
Strategic	Group, *F*(1, 85) = 21.02, *p* < .001, η_p_^2^ = .20 Dialect, *F*(1, 85) = 70.17, *p* < .001, η_p_^2^ = .45			
Modified	NA			

*Note.* AAE = African American English; SWE = Southern White English; SLI = children with specific language impairment; TD = typically developing children; NA = not applicable.

#### Auxiliary BE Present

All four child groups again produced all form types to express auxiliary BE present (see Figures 4a and 4b). Proportions of mainstream overt forms were highest for *is*, and zero forms were highest for *are,* with both SLI groups producing more zero forms than the TD groups. Nonmainstream overt forms were infrequent; of the 35 produced, 22 involved *is* produced with a second-person singular (e.g., *You's going to do it*) or plural subject (e.g., *Them two boys is punching, Those is sleeping*), and 13 involved *is* or *are* produced within a noninverted *wh*-question (e.g., *What this is, What you're recording, Why it's doing that, When he's gonna do a rescue, Where the fish is gonna go, How the dad is going to sit on here*), with a few of these forms in noninverted questions also containing an auxiliary *is* with a plural subject (e.g., *What they's bringing to the picnic*; *Why you's not coming*). As before, forms classified as other were rare (e.g., *He didn't know what is he doing, He's tows stuff*).

Using the 87 (82%) samples containing at least eight or more BE present forms for the analyses, both the unmodified and strategic scoring approach led to clinical group and dialect differences, and the effect sizes of the differences were the same (clinical group: η_p_^2^ = .09; dialect: η_p_^2^ = .24; see Table 8). As before, statistical analyses were not applied to percentages from the modified scoring approach because of ceiling effects (all group *M*s ≥ 99%).

**Table 8.** Mean (*SD*) percent of marking auxiliary BE present by dialect, clinical status, and scoring approach (*n* = 87).

Variable	AAE		SWE	
	SLI (*n* = 32)	TD (*n* = 30)	SLI (*n* = 13)	TD (*n* = 12)
Scoring approach				
Unmodified	26 (22)	36 (31)	50 (24)	73 (20)
Strategic	28 (22)	38 (30)	52 (26)	75 (19)
Modified	100 (02)	100 (01)	99 (02)	100 (00)
Significant effects				
Unmodified	Group, *F*(1, 83) = 8.01, *p* = .006, η_p_^2^ = .09 Dialect, *F*(1, 83) = 25.71, *p* < .001, η_p_^2^ = .24			
Strategic	Group, *F*(1, 83) = 7.73, *p* = .007, η_p_^2^ = .09 Dialect, *F*(1, 83) = 26.56, *p* < .001, η_p_^2^ = .24			
Modified	NA			

*Note.* AAE = African American English; SWE = Southern White English; SLI = children with specific language impairment; TD = typically developing children; NA = not applicable.

#### Auxiliary BE Past

All four child groups again produced all form types to express auxiliary BE past (see Figures 5a and 5b). Proportions of mainstream overt forms were highest for *was* contexts, and proportions of nonmainstream overt forms were highest for *were* contexts. Of the nonmainstream overt forms, 165 involved *was* with a second-person singular (e.g., *You was fixing it?*) or plural subject (e.g., *They was washing a car, The oranges was dropping*), and four involved *were* with a third-person singular subject (e.g., *And Benjamin were watching them*). Unlike the other T/A structures, *was* and *were* forms were not found in noninverted *wh*-questions. Zero forms also were infrequent (e.g., *Then the car Ø coming and then he died, He Ø fixing to fall and I caught him*), and only one form classified as other was found in the samples (i.e., *Then them was started picking lemons*).^6^

Only 62 (58%) samples contained at least eight coded BE past forms, so the number of samples analyzed for this structure was much lower than the other structures and the results should be viewed with caution, especially for the dialect of SWE. As shown in Table 9, the unmodified approach led to null findings, and the modified approach led to ceiling effects (all group *M*s = 100%). The strategic scoring approach showed a dialect (AAE < SWE) difference; however, for both dialects, percentages of marking were above 90% (AAE *M* = 95, *SD* = 07; SWE *M* = 99, *SD* = .02), and the effect size of the dialect difference was small (η_p_^2^ = .08).

**Table 9.** Mean (*SD*) percent marking of auxiliary BE past by dialect, clinical status, and scoring approach (*n* = 62).

Variable	AAE		SWE	
	SLI (*n* = 21)	TD (*n* = 27)	SLI (*n* = 5)	TD (*n* = 9)
Scoring approach				
Unmodified	77 (13)	77 (12)	83 (26)	86 (07)
Strategic	94 (08)	96 (07)	100 (00)	99 (02)
Modified	100 (00)	100 (00)	100 (00)	100 (00)
Significant effects				
Unmodified	Null findings			
Strategic	Dialect, *F*(1, 58) = 5.28, *p* = .025, η_p_^2^ = .08			
Modified	NA			

*Note.* AAE = African American English; SWE = Southern White English; SLI = children with specific language impairment; TD = typically developing children; NA = not applicable.

### Cut Scores and Classification Accuracies of the T/A Structures Using Strategic Scoring

Given the earlier results, the final analysis examined the classification accuracy of the four T/A structures using the strategic scoring approach. These analyses were completed twice, once with only the samples that contained at least eight coded forms and once with all samples. The results were the same for past tense as all samples contained at least eight coded forms, but for the other structures, accuracy was slightly better with the samples that contained at least eight forms. Cut scores for these other structures also changed, depending on the number of samples included in the analyses. Perhaps most importantly, even when the analyses were restricted to the samples with at least eight forms, measures of past tense yielded the highest levels of SLI versus TD classification accuracy. With the dialects combined and with a past tense cut score between 82.15% and 82.19%, classification accuracy was 77%, Sp = .70, Sp = .85. With the dialects separated, the AAE cut score changed to 80.01%–81.24% (classification accuracy = 77%, Se = .70, Sp = .85), and the SWE cut score changed to 84.86%–86.48% (classification accuracy = 75%, Se = .61, Sp = .89). As was found for the structures combined, when dialect-specific cut scores and classification accuracies were calculated by hand using the maximum of Youden's *J* index (instead of within a discriminant analysis), even better outcomes were obtained (i.e., best AAE cut score was between 82% and 83%, classification accuracy = 80%, Se = .83, Sp = .77; best SWE cut score was between 91% and 93%, classification accuracy = 89%, Se = .89, Sp = .89).

## Discussion

Using a *disorder within dialects* framework, a small but growing number of AAE and SWE studies have revealed reliable differences between children with and without SLI in the percentages at which T/A structures are overtly marked. The most recent evidence to support this claim was reported by Oetting et al. (2019) in a study of four dialect-informed probes. In the current study, we examined how well findings from the probes generalize to language samples. Although low numbers of some forms limited the analyses, the results generally supported those found for the probes. Within the samples, the children produced a large and diverse inventory of mainstream and nonmainstream T/A forms; a strategic scoring approach led to the largest differences between the clinical groups while reducing differences between the children's dialects; dialect-specific cut scores performed better than dialect-universal cut scores, especially when these were calculated using the maximum of Youden's *J* index; and of the four T/A structures, measures of past tense led to the highest levels of within-dialect SLI versus TD classification accuracy.

### Other Comparisons Between the Samples and Probes

Although the language samples and dialect-informed probes resulted in similar findings, there are other comparisons between these two tasks that need to be considered before recommending probes for clinical practice. As noted in the introduction, language samples are extremely time-consuming to elicit, transcribe, code, and analyze, yet if they provide useful information about children's T/A systems that cannot be collected with probes, then language samples may remain the preferred task within clinical practice.

Regarding the forms elicited, the children produced the same mainstream and nonmainstream overt forms and zero forms elicited by the probes within their language samples, as well as some other nonmainstream overt forms that were not elicited by the probes. The latter included dialect-specific forms for past tense irregular (e.g., *brung*); use of verbal –*s* forms with first-person singular, second-person singular, and plural subjects (e.g., *The red lights means stop*); and use of past tense, verbal –*s, is*, and *are* forms within noninverted *wh*-questions (e.g., *Where you went*; *Where this goes*; *What this is?*). The nature of the probes did not allow for these types of forms to be elicited. Using a set of probes instead of language samples would lead to a loss of information about these forms; however, these forms represented less than 1% of the coded forms within the samples.

The two tasks also generated different types of forms classified as other. These forms represented 8% of the probe data and involved responses without clausal structure to support the targeted T/A form, whereas they represented 1% of the sample data and involved use of an overt T/A form in a dialect-inappropriate context. The strategic scoring approach excludes all forms classified as other, so if strategic scoring is implemented, then this difference between the two tasks becomes less of a concern. Nevertheless, using a set of probes instead of language samples would lead to a loss of information about dialect-inappropriate form use (but one would gain information about children's abilities to produce clausal structure within a structured task—information that may be more applicable to a child's use of language in other school-based tasks).

Perhaps more importantly, the samples and probes generated the same dialect-specific patterns of overt marking for the individual T/A structures. Within the samples and with strategic scoring, the AAE TD group produced a higher percentage of overt marking for past tense (88%) than for verbal –*s* (32%), a higher percentage for BE past (96%) than for BE present (38%), and a higher percentage for *is* (46%) than for *are* (28%). By comparison, the SWE TD group produced high percentages of overt marking for past tense (96%), verbal –*s* (85%), and BE past (99%) and a lower rate for BE present (75%), with the lower percentage of the latter tied to the children's marking of *are* (*is* = 94% vs. *are* = 47%). The AAE and SWE SLI groups also showed similar patterns of overt marking for the individual T/A structures within the language samples, although differences between the structures were less dramatic given their overall lower percentages of overt marking: AAE SLI: past tense (73%) versus verbal –*s* (18%), BE past (94%) versus BE present (28%), *is* (31%) versus *are* (21%); SWE SLI: past tense (74%), verbal –*s* (52%), and BE past (100%) versus BE present (52%), *is* (66%), versus *are* (38%). These results are consistent with the probe data and the adult AAE and SWE literature and suggest that either samples or probes could be used to learn about AAE- and SWE-speaking children's patterns of T/A marking.

With strategic scoring, the probes also showed a clinical group effect for all four T/A structures, whereas the language samples showed this effect for three (i.e., past tense, verbal –*s,* auxiliary BE present). Effect sizes of the clinical group differences were also largest with the probes (i.e., structures combined: probes η_p_^2^ = .38 vs. samples η_p_^2^ = .30; structures separated: probes η_p_^2^ ranged from .17 to .40 vs. samples η_p_^2^ ranged from .05 to .30). A different pattern was observed for the children's dialects. The samples showed dialect effects for four structures (i.e., past tense, verbal –*s,* BE present, and BE past), whereas the probes showed this effect for only two (i.e., past tense, verbal –*s*). Effect sizes of the dialect differences were also largest within the samples (i.e., structures combined: samples η_p_^2^ = .18 vs. probes η_p_^2^ = .07; structures separated: samples η_p_^2^ ranged from .04 to .45 vs. probes η_p_^2^ ranged from .08 to .18). Taken together, these findings indicate that the probes were more sensitive to the clinical status of the children and the language samples were more sensitive to dialect differences between the children.

Finally, all children were included within the analyses of the probes, except for five who lacked scorable responses for the BE past probe. In sharp contrast, many children had to be excluded from the analyses of the language samples because of lack of data. The nature of the probe data also allowed for the eight T/A structures to be treated as separate variables within the same discriminant function, and this led to a higher classification accuracy than when these structures were combined. The low number of coded forms for some of the T/A structures within the language samples did not allow for this same analysis nor a statistical analysis of the eight different T/A structures. This result is noteworthy because the samples averaged over 200 analyzed utterances, which is larger than the approximately 100 analyzed by Rudolph et al. (2019) and the 50- to 100-utterance criterion that is typically recommended for clinical practice. Samples with no forms for a T/A structure cannot be analyzed, and samples with only a few forms limit one's ability to detect individual differences among children. Moreover, whereas language samples take 20–30 min to elicit and hours to transcribe and code, each 16-item probe can be administered in 5–10 min, and scoring can be completed during administration. Clinicians need tools that lead to sufficient amounts of data that are also quickly and easily administered and scored. Dialect-informed probes meet this clinical need, whereas language samples do not.

### Comparison of Findings to Other Studies

Recall that Rudolph et al. (2019) argued against measures of T/A when working with groups of diverse English dialect speakers. Their conclusions were based on measures of slightly different T/A structures, and some of their language samples contained very low numbers of T/A forms in comparison to the samples used in this study. They also did not consider differences in the children's marking of the various T/A structures, which, as we have shown here and elsewhere, differ across dialects, nor did they strategically score mainstream and nonmainstream overt forms that are part of many dialects of English. Finally, Rudolph et al. did not consider the children's type of dialect but, instead, used a single cut score of 85% from the dialect of GAE. In the current study and using the Youden's *J* index method, the best cut score for the T/A structures combined was 60% for AAE and 80% for SWE. A comparison of the two studies is not intended to be a recommendation for a specific set of T/A structures or cut scores. Instead, it is offered to highlight the importance of understanding and taking into account the dialects of the children being evaluated, and this includes knowing frequency-based information about how various T/A structures are marked within dialects and collecting a sufficient amount of data to capture these frequency-based differences so that T/A structures can be measured in a dialect-appropriate manner.

Although we disagree with Rudolph et al.'s (2019) conclusions, our findings do caution against composite measures of T/A without appropriate reporting of the data included within these measures. Forty-nine percent of the coded forms studied here were for past tense, which means that the percentages calculated for the T/A structures combined were heavily weighted by these forms. Had the language samples elicited conversations about the present or future, or even the relative past, the distributions of the coded forms for each T/A structure would have differed, and this would have yielded very different percentages and cut scores for the T/A structures combined, especially for the dialect of AAE.

The relative frequencies of the coded forms for each structure also differed by the children's dialects and clinical status. Thus, the combined T/A percentages for the four groups studied here reflected different relative distributions of the T/A structures. This finding has implications for studies of not only AAE and SWE but also for other dialects, including GAE. If same dialect–speaking children with and without SLI do not produce the same relative frequencies of each T/A structure, then their corresponding composite measures of T/A will not be equivalent. Reporting frequency information as shown in Table 5 for each structure, dialect, and clinical group would offer a much-needed level of transparency whenever composite measures of T/A structures are included within a study. As an alternative, use of dialect-informed probes instead of samples to calculate percentages of T/A form use allows for control over the types and frequencies of the structures elicited.

Beyond Rudolph et al. (2019), the results of this study and the probe study complement and extend other studies conducted on TD children who speak rural and urban varieties of AAE and TD children who speak rural varieties of SWE (for a review, see Oetting, 2019). Most of the previous studies have focused on one T/A structure or a small set of structures (e.g., copulas were studied by Wyatt, 1996; past tense was studied by Lee & Oetting, 2014), although a few have examined two or three structures together. For example, verbal –*s* and progressive –*ing* were studied by Newkirk-Turner and Green (2016); past tense, verbal –*s*, and plurals were studied by Hendricks and Adlof (2020); and past tense was studied and compared to verbal –*s* by Green (2019). In one study, Seymour et al. (1998) examined 17 structures but used an unmodified scoring approach only. The current study examined both multiple structures and scoring approaches. In addition, not all of these studies have categorized children's forms as mainstream overt, nonmainstream overt, or nonmainstream zero, but of those that have, all three types have been found in the children's grammars when the method of elicitation provided an appropriate context for them. The current study considers all three of these forms as well as forms classified as other and their relative frequencies as a function of the children's dialects and T/A structure.

Studies examining how TD children use various forms to express T/A (and other aspects of their linguistic systems) within AAE and SWE are accumulating. Findings from these studies need to be incorporated into descriptions of child AAE and SWE within textbooks and clinical resource manuals. For the dialect of AAE, children's grammars are often described as allowing optional marking or variable marking, with examples limited to mainstream overt and nonmainstream zero forms (e.g., *walked* vs. *walkØ*, *he is running* vs. *he Ø running*). As shown in the current study and elsewhere, evidence indicates that this type of singular statement about the optionality of child AAE is both too restrictive (because it does not capture the full inventory of T/A forms found in children's dialects) and too general (because it does not describe important differences in the frequencies at which children produce their various forms by type of T/A structure). Richer descriptions of child AAE and SWE (and other dialects) that highlight the large and diverse inventory of forms children use along with frequency-based information about form use across structures (and across tasks, modes of communication, audiences, settings) would help speech-language professionals better understand these dialects and others.

The current findings are also consistent with many GAE studies that have found T/A deficits to be part of the SLI grammar profile (Rice, 2003; Rice & Wexler, 1996; Rice et al., 1998). Specifically, the nature of the SLI deficit in GAE is one of lower-than-expected percentages of T/A overt forms, along with relatively error-free use of overt forms when language samples are examined. GAE-speaking children with SLI also underproduce utterances supporting T/A structures relative to same dialect–speaking TD controls. These three patterns of results were also documented here, because the children with SLI produced lower-than-expected percentages of overt forms for their dialects, very low levels of dialect-inappropriate errors, and fewer utterances to support T/A structures than the TD controls, even though their samples contained more utterances. Overlap in findings allows for cross-dialect generalizations to be made about the SLI grammar profile, with the added caveat that measures of T/A must use (a) dialect-appropriate materials, coding, and scoring; (b) dialect-specific (and perhaps community-specific) cut scores based on comparisons of same dialect–speaking TD children; and (c) enough data to capture clinical group differences.

A final caveat relates to the magnitude of the T/A clinical effects across dialects. The effect size of the SLI versus TD difference was larger in SWE than in AAE for some of the structures (and for the general measure of MLU). A similar result was found in the probe study. Also, although measures of past tense yielded the largest clinical differences within the language samples and probes, the magnitude of the clinical effects was not as large as reported in a meta-analysis of SLI studies conducted in studies of other English dialects and non-English Germanic languages/dialects (Krok & Leonard, 2015). Effect size differences across dialects and languages are expected given differences across linguistic systems. For now, though, the more important point is that there is mounting evidence showing T/A deficits to be part of the SLI grammar profile in children who speak either AAE or SWE.

### Clinical Implications

T/A structures should be assessed in AAE- and SWE-speaking children with SLI using dialect-appropriate materials, coding, and scoring, and these structures should be treated when children with SLI produce lower percentages of overt forms relative to their same dialect–speaking TD peers. Regarding assessment, a 90% classification accuracy level is often considered the gold standard for using a measure to identify a child as SLI or TD (Dollaghan, 2007).^7^ Measures of T/A within the language samples (and probes) came close but did not meet this high level of accuracy (e.g., strategic scoring of past tense in the current study with Youden's *J*: accuracy = 80% in AAE and 89% in SWE). Thus, regardless of task, these findings indicate that it is important to combine measures of T/A with other measures to identify children with SLI or use them descriptively to profile a child's linguistic strengths and weaknesses relative to same dialect–speaking TD peers. This recommendation is no different than the one we would offer clinicians working with GAE-speaking children, because expressions of T/A, while critically important for communication, represent a subset of skills children need to succeed socially and academically. Other tools to consider with kindergartners from diverse dialect groups include the Diagnostic Evaluation of Language Variation–Norm Referenced, a parent and teacher interview or questionnaire, a brief standardized language sample, classroom observation, and dynamic assessment (Hyter & Salas-Provance, 2019).

Regarding treatment, possible goals for AAE- and SWE-speaking children with SLI include increasing their use of clausal structure within utterances to support T/A forms and increasing children's use of various dialect-appropriate T/A forms to match the frequency distributions of their dialect community. Ideally, goals targeting conversational speech would not include statements about *use with 80% or 90% accuracy*; these levels of use and the very term *accuracy* are tied to mainstream form use in the dialect of GAE and are not appropriate for other dialects of English.

Goals focused on mainstream overt form accuracy also do not necessarily lead to treatments that help children with SLI (whether they speak AAE, SWE, or GAE) develop grammatically rich and flexible systems. Fortunately, grammar interventions have progressed. Literature can now be found to support treatment goals focused less on accuracy and more on sentence diversity (e.g., Hadley, 2014; Hadley et al., 2018) and complex syntax diversity (e.g., Weiler & Schuele, 2014). Following these approaches, treatments would seek to increase children's use of many different dialect-appropriate T/A forms within many different types of sentences (e.g., those with a variety of subjects, main verbs, secondary verbs, and independent and dependent clauses), modes of communication (e.g., speaking, reading out loud, writing, or texting), and goals of communication (e.g., to ask or answer questions, share opinions, retell events, critique, persuade, and entertain). Treatments focused on increasing children's metalinguistic abilities of grammar are also increasing in the field (e.g., Balthazar et al., 2020). Focusing on diversity and flexibility of language and metalinguistics abilities within grammar treatments align well with both common core standards and regular education approaches that affirm students' rights to their own language in school (Scott et al., 2009; for resources on teaching linguistic versatility and other culturally sustaining teaching practices, see many chapters in Blake & Buchstaller, 2020; Charity Hudley & Mallinson, 2014; Young et al., 2014).

### Limitations and Future Directions

The language samples were limited, in that they came from kindergartners who lived in one community in the rural South. Not all communities of AAE and SWE child speakers will produce every T/A form documented here or produce the same frequencies of these forms. More studies are needed to document the T/A forms and frequencies of children who speak other varieties of AAE and SWE and other dialects of English. Given the developmental changes that occur in children's T/A systems, studies of younger and older AAE- and SWE-speaking children are also needed.

It is likely premature to apply the cut scores identified in the current study to other groups of children without caution, but research and clinical efforts should be directed toward testing these cut scores and different cut scores. Recall also that we identified different cut scores and achieved higher levels of classification accuracy for the T/A structures combined and for past tense using Youden's *J* indices rather than discriminant analyses. Youden's *J* index (Youden, 1950) has been used elsewhere within SLI studies employing receiver operating characteristic curve analyses (e.g., Redmond et al., 2019), and future studies may want to compare the optimal cut scores from Youden's *J* index to the classifications resulting from discriminant analysis. Future studies should also explore different strategic scoring approaches and cut scores for different types of tasks and grammatical structures.

Finally, paradigm shifts are always difficult, and learning how to measure and treat the T/A deficits of children with SLI across dialects and in dialect-appropriate ways is no exception. The current study narrowly focused on children's grammars but not on the broader topic of culturally responsive practice. Future studies are needed to improve the field's ability to engage in culturally responsive practices, and this includes not only our behaviors within assessment and treatment but also the ways we describe other dialects than GAE and the dialect of GAE and advocate for the small percentage of children with SLI within all dialects and cultures who struggle to learn language commensurate with their same dialect–speaking peers (for resources, see Charity Hudley et al., 2018; Hamilton et al., 2018; Hyter & Salas-Provance, 2019).

## Conclusions

Findings previously reported for a set of dialect-informed probes are supported by analyses of language samples. Given this, T/A structures should be assessed in AAE- and SWE-speaking children with SLI and treated when these children produce lower percentages of overt forms relative to those of same dialect–speaking TD peers. When measuring and treating T/A structures, it is critical to use dialect-appropriate methods. These methods include evaluating a wide range of T/A structures, collecting enough data to evaluate the many types of forms children use to express T/A, and implementing a strategic scoring approach and dialect-specific cut scores. A comparison of the findings from the samples and probes also indicates that dialect-informed probes are the better task for identifying the T/A deficits of children with SLI in AAE and SWE. Whether one uses probes or samples, unmodified scoring approaches that count all nonmainstream forms as incorrect and modified scoring approaches that count all nonmainstream forms as correct without considering their frequencies are not recommended.

## Supplementary Material

10.1044/2020_JSLHR-20-00243SMS1 Supplemental Material S1 Examples of regular past tense form types and scoring approaches.

10.1044/2020_JSLHR-20-00243SMS2 Supplemental Material S2 Supplemental analyses.

## Footnotes

1 In AAE and SWE, a speaker's use of various mainstream and nonmainstream overt forms and zero forms to express T/A also varies by other variables (i.e., age, task, audience) unrelated to the current study.

2 The TEGI is an exception to other GAE-centric tests; this tool treats overregularized past tense forms (e.g., *catched*) as overtly marked.

3
Rice and Wexler (2001) specifically note that the TEGI (and by extension cut scores from the TEGI) was designed for “children who speak Standard American English” (p. 55). Thus, these authors never recommended a single cut score for T/A marking for children who speak diverse dialects of English. Gladfelter and Leonard (2013), although they did not classify their participants' dialects as GAE, provided race, community, and grammar test information about their participants that could have been used by Rudolph et al. (2019) to render an 85% cut score as inappropriate for their Pittsburg sample.

4 Sample size differences between the SLI and TD groups were explored by comparing the number of utterances each child produced in the random 1-min excerpts from the listener judgment task; the number of utterances within the SLI and TD excerpts did not differ (*M* ~ 11 utterances). We also checked the length of the sessions that corresponded to the transcribed utterances in the longest and shortest 10 samples from each group (*n* = 40). The sessions of the longest SLI samples averaged 4 min longer than the TD samples, and the sessions of the shortest SLI samples averaged 7 min longer than the TD samples. The examiners were blind to the clinical status of the children during language sample collection and transcription, so the sample differences were unintentional.

5 Production of *is* with a second-person or plural subject was coded as a nonmainstream form of *are,* and production of *are* with third-person singular subjects was coded as a nonmainstream form of *is*. This decision was based on coding from the probe study (Oetting et al., 2019; see also Berry & Oetting, 2017). The same coding decisions were made for *was* and *were*. In most studies of language samples, *is, are, was*, and *were* forms are coded as produced (e.g., *is* gets coded as a form of *is*), regardless of the subject of the predicate. Also, for verbal *–s*, all forms classified as nonmainstream overt and other were classified as encoding a nonhabitual meaning, because our coding was unreliable when we attempted to assign a ± habitual meaning to these forms. None of these coding decisions affected the findings because the structures were combined within the statistical analyses.

6 We checked the pausing of this utterance to rule out a revision of *was* for *started*; however, although we are presenting this utterance as a *was* form classified as other, it could be classified as a regular past tense form classified as other. Indeed, it was coded both ways by different examiners. The important point is that these other T/A forms are rare in the language samples.

7
Youngstrom (2014) suggests a lower standard of accuracy for clinical conditions, but 90% is maintained here for sake of argument.
