# Supplementary material for: Marking of Tense and Agreement in Language Samples by Children With and Without Specific Language Impairment in African American English and Southern White English: Evaluation of Scoring Approaches and Cut Scores Across Structures
Source: J Speech Lang Hear Res. 2021 Jan 20;64(2):491–509. doi: 10.1044/2020_JSLHR-20-00243 (PMC8632490; doi:10.1044/2020_JSLHR-20-00243)
Supplement: Supplemental Material S1 [file JSLHR-64-491-s001.pdf]

Supplemental material, Oetting et al., "Marking of Tense and Agreement in Language Samples by Children With and Without Specific Language Impairment in African American English and Southern White English: Evaluation of Scoring Approaches and Cut Scores Across Structures," *JSLHR*, https://doi.org/10.1044/2020_JSLHR-20-00243

Supplemental Material S1. Examples of regular past tense form types and scoring approaches.

AAE-speaking child classified as SLI #967

Regular Past Tense Form Types		
Type of Form	Frequency	Verb
Mainstream Overt	3	bump/ed, bump/ed, bump/ed
Nonmainstream Overt	0	--
Nonmainstream Zero	7	bumpØ, bumpØ, washØ, dieØ, openØ, pooØ, finishØ
Other	1	...gonna bump/ed his head.
Scoring Approach		
Unmodified<br>Modified<br>Strategic	3/(3 + 0 + 7 +1) = 27%<br>(3 + 0 + 7)/(3 + 0 + 7 + 1) = 91%<br>(3 + 0)/(3 + 0 + 7) = 30%	

AAE-speaking children classified as TD #715

Regular Past Tense Form Types		
Type of Form	Frequency	Verb
Mainstream Overt	13	want/ed, happen/ed, pee/ed, poo/ed, poo/ed, mix/ed, happen/ed, hate/ed, mess/ed, mess/ed, start/ed, ask/ed, start/ed
Nonmainstream Overt	13	had poo/ed, had back, had die/ed, had laugh, had whoop/ed, had drop/ed, had die/ed, had step/ed, had die/ed, had jump/ed, had punch, had punch/ed, had die/ed,
Nonmainstream Zero	3	stopØ, tripØ, bustØ
Other	0	--
Scoring Approach		
Unmodified<br>Modified<br>Strategic	13/(13 + 13 + 3 + 0) = 45%<br>(13 + 13 + 3)/(13 + 13 + 3 + 0) = 100%<br>(13 + 13)/(13 + 13 + 3) = 90%	

SWE-speaking children classified as SLI #781

Regular Past Tense Form Types		
Type of Form	Frequency	Verb
Mainstream Overt	3	scare/ed, bump/ed, happen/ed
Nonmainstream Overt	1	had happen/ed
Nonmainstream Zero	4	wantØ, popØ, popØ, happenØ
Other	1	every day we use/ed (context: every day we use)
Scoring Approach		
Unmodified<br>Modified<br>Strategic	3/(3 + 1 + 4 + 1) = 33%<br>(3 + 1 + 4)/(3 + 1 + 4 + 1) = 89%<br>(3 + 1)/(3 + 1 + 4) = 50%	

SWE-speaking child classified as TD #888

Regular Past Tense Form Types		
Type of Form	Frequency	Verb
Mainstream Overt	17	help/ed, stop/ed, close/ed, pass/ed, walk/ed, turn/ed, potty/ed, turn/ed, punch/ed, happen/ed, scream/ed, need/ed, want/ed, ask/ed, ask/ed, step/ed, trip/ed
Nonmainstream Overt	1	had punch/ed
Nonmainstream Zero	0	--
Other	0	--
Scoring Approach		
Unmodified<br>Modified<br>Strategic	17/(17 + 1 + 0 + 0) = 94%<br>(17 + 1 + 0)/(17 + 1 + 0 + 0) = 100%<br>(17 + 1)/(17 + 1 + 0) = 100%	
