# Supplementary material for: Marking of Tense and Agreement in Language Samples by Children With and Without Specific Language Impairment in African American English and Southern White English: Evaluation of Scoring Approaches and Cut Scores Across Structures
Source: J Speech Lang Hear Res. 2021 Jan 20;64(2):491–509. doi: 10.1044/2020_JSLHR-20-00243 (PMC8632490; doi:10.1044/2020_JSLHR-20-00243)
Supplement: Supplemental Material S2 [file JSLHR-64-491-s002.pdf]

Supplemental Material S2. Supplemental analyses.

We analyzed our data in two ways: 1) using a 2 × 2 ANOVA for each dependent variable as was done with the Oetting et al. (2019) probe data from the same children, and 2) using a logistic regression with a binomial distribution on the proportions calculated with the unmodified and strategic scoring approaches. As with ANOVA, what enters the numerator and denominator of the proportions varied by definition of the unmodified and strategic scoring approach. We calculated the likelihood ratio Chi-squared for both the main effects of group and dialect and their interactions.

The logistic regression has the advantage of not having to conform to the assumptions of normality and homogeneity of variance of the ANOVA. While the homogeneity of variance assumption is not a large concern when groups are equal (as was our case for the group variable—there were 53 children in both the SLI and TD groups), it can lead to a lower ability to find differences if the larger group shows higher variability—which would be the case if AAE speakers (n = 70) had higher variability than SWE speakers (n = 36). Thus, the logistic regression may be able to detect some significant findings relevant to the dialect factor or its interaction that the ANOVA was not able to detect due to violations of homogeneity of variance.

In many cases the two analyses showed similar results; but on some occasions, the logistic regression found an additional significant effect of the interaction between group and dialect. In each of these cases, the groups continued to differ within each dialect, but the effect was larger in the SWE speakers than the AAE speakers. Finding larger clinical group effects in SWE than in AAE was also found with some of the ANOVAs, and it has been found in most of our previous studies with these same participants (McDonald & Oetting, 2019; McDonald et al., 2018; Oetting et al., 2019).

Statistics for the logistic regression

<u>All structures considered together</u>

	Chi Squared	*df*	*p* ≤
Unmodified scoring—same as ANOVA			
Group	456.947	1	.001
Dialect	330.222	1	.001
Interaction	74.971	1	.001
Strategic scoring—additional finding of an interaction that had been at .06 in the ANOVA			
Group	466.909	1	.001
Dialect	218.078	1	.001
Interaction	91.342	1	.002

<u>Past tense</u>

Unmodified scoring—additional finding of an interaction that at been at .16 in the ANOVA			
Group	195.131	1	.001
Dialect	118.212	1	.001
Interaction	27.030	1	.001

Strategic scoring—additional finding of an interaction that at been at .15 in the ANOVA

Group	208.087	1	.001
Dialect	27.442	1	.001
Interaction	27.342	1	.001

Third person singular

Unmodified scoring—same as ANOVA

Group	204.624	1	.001
Dialect	184.390	1	.001
Interaction	10.792	1	.001

Strategic scoring—additional finding of an interaction that had been at .06 in the ANOVA

Group	199.074	1	.001
Dialect	223.941	1	.001
Interaction	14.925	1	.001

BE present

Unmodified scoring—same as ANOVA

Group	37.702	1	.001
Dialect	146.313	1	.001
Interaction	2.014	1	156

Strategic scoring—same as ANOVA

Group	37.089	1	.001
Dialect	145.706	1	.001
Interaction	2.346	1	.126

BE past

Unmodified scoring—same as ANOVA

Group	.246	1	.620
Dialect	2.914	1	.088
Interaction	.291	1	.589

Strategic scoring—same as ANOVA

Group	.807	1	.369
Dialect	6.994	1	.008
Interaction	1.586	1	.208
